# Relationships of eeg and immunological parameters in depressive patients who survived COVID-19

**DOI:** 10.1192/j.eurpsy.2023.738

**Published:** 2023-07-19

**Authors:** A. F. Iznak, E. V. Iznak, E. V. Damyanovich, S. A. Zozulya, I. V. Oleichik

**Affiliations:** 1 Laboratory of Neurophysiology; 2 Laboratory of Neuroimmunology; 3 Department of endogenous disorders, Mental Health Research Centre, Moscow, Russian Federation

## Abstract

**Introduction:**

Coronavirus infection affects the CNS and modulates the immune system. The associated processes of neuroplasticity play an important role in the pathogenesis of depression.

**Objectives:**

The aim of the study is to identify the relationships between EEG and immunological parameters in depressive patients who recovered from coronavirus infection, in order to clarify the features of neuroimmune interaction after suffering COVID-19.

**Methods:**

30 female patients aged 16-25 enrolled in the study were admitted for treatment during the pandemic in 2020-2022 (“COVID” group). Previously, they had been ill with COVID-19 in a mild or asymptomatic forms on a background of depressive state (F31.3-4, F21.3-4 + F34.0, according to ICD-10) from 3 months to 2 years before the examination.

All patients underwent pre-treatment multichannel background EEG recordings in a state of quiet wakefulness with eyes closed and analysis of the absolute EEG spectral power (SP) in 8 narrow frequency sub-bands.

As well, markers of neuroplasticity ─ the levels of autoantibodies to the S100b protein (AAT-S100b) and to the myelin basic protein (AAT-MBP) were measured in each patient’s blood plasma using the laboratory technology “Neuro-immuno-test”.

The EEG and immunological parameters of the “COVID” group were compared with similar data of 40 depressive patients who were treated in 2018-2019, that is, they did not have COVID-19 (“pre-COVID” group), but matched by sex, age, syndrome structure, as well as the pre-treatment severity of depression (according to the HDRS-17 scale) to patients of the “COVID” group.

Statistical analysis of the data obtained was carried out by the correlation analysis method of the IBM SPSS Statistics, v.22 software package.

**Results:**

In the “COVID” group, the AAT-S100b level values positively correlated with the EEG delta sub-band (2-4 Hz) SP values in T3, T4, P4, and O1 leads. The values of the AAT-MBP level correlated with the SP values of delta (2-4 Hz) and theta1 (4-6 Hz) EEG sub-bands in C3, T4, P3, P4, O1, and O2 leads. In the “pre-COVID” group, the values of the AAT-S100b level correlated positively with the SP values of not slow-wave, but alpha2 (9-11 Hz) and alpha3 (11-13 Hz) EEG activity in T3, P3, O1, and O2 leads.

**Conclusions:**

Positive correlations of the AAT-S100b level with alpha2 and alpha3 SP values indicate that in the “before COVID” group, the AAT-S100b level reflects rather the reparative processes of neuroplasticity. On the contrary, in the “COVID” group, positive correlations of the AAT-S100b and of the demyelination marker AAT-MBP levels with the SP values of slow-wave (delta and theta1) EEG frequency components, reflecting a reduced brain functional state, indicate that elevated levels of AAT-S100b and AAT-MBP in this group are markers of nerve tissue damage caused by coronavirus infection.

The study supported by the Russian Science Foundation (grant No. 21-18-00129).

**Disclosure of Interest:**

None Declared

